# Lipoperoxidation and Protein Oxidative Damage Exhibit Different Kinetics During Septic Shock

**DOI:** 10.1155/2008/168652

**Published:** 2008-06-16

**Authors:** Max Andresen, Tomas Regueira, Alejandro Bruhn, Druso Perez, Pablo Strobel, Alberto Dougnac, Guillermo Marshall, Federico Leighton

**Affiliations:** ^1^Departamento de Medicina Intensiva, Facultad de Medicina, Pontificia Universidad Católica de Chile, Alameda, 340 Santiago, Chile; ^2^Departamento de Biología Celular y Molecular, Facultad de Ciencias Biológicas, Pontificia Universidad Católica de Chile, Alameda, 340 Santiago, Chile; ^3^Facultad de Matematicas, Pontificia Universidad Católica de Chile, Alameda, 340 Santiago, Chile

## Abstract

Septic shock (SS)-related multiorgan dysfunction has been associated with oxidative damage, but little is known about the temporal damage profile and its relationship to severity. The present work investigated prospectively 21 SS patients. Blood samples were obtained at diagnosis, 24, 72 hours, day 7, and at 3 months. At admission, thiobarbituric acid reactive substances (TBARSs), plasma protein carbonyls, plasma protein methionine sulfoxide (MS), ferric/reducing antioxidant power (FRAP), total red blood cell glutathione (RBCG), uric acid (UA), and bilirrubin levels were increased (*P* < .05). Total radical—trapping antioxidant potential (TRAP) and vitamin-E were similar to controls, and vitamin-C was decreased (*P* < .05). During evolution, TBARS and RBCG increased (*P* < .001), vitamin-E levels remained stable, whereas plasma protein carbonyls and MS, TRAP, vitamin-C, reduced glutathione, and UA levels decreased (*P* < .006). After 3 months, plasma protein carbonyls and MS persisted elevated. More severe patients exhibited higher TBARS, TRAP, FRAP, vitamin-C, UA, and bilirrubin levels. Our results suggest early and persistent oxidative stress during septic shock and a correlation between increasing levels of lipoperoxidation and sepsis severity.

## 1. INTRODUCTION

Inflammation, sepsis, and particularly septic shock are associated with global and local hypo-perfusion, ischemia-reperfusion events, endothelial injury with an associated procoagulant state, and monocyte—macrophage system activation. All these processes contribute to the production and release of different cytokines and other inflammatory mediators [1]. They also induce the production of large amounts of free radicals in a nonregulated fashion associated with high-oxidative potential damage [2]. In fact, several sources of reactive oxygen species (ROS) have been detected in sepsis and septic shock, including the mitochondrial respiratory electron transport chain, immune cell, and xanthine oxidase activation as a result of ischemia and reperfusion and the respiratory burst associated with NADPH oxidase [3].

Several studies have shown the presence of oxidative stress in sepsis [4–9]. Early production of reactive oxygen species (ROS) has been demonstrated in experimental studies in sepsis [10], and excessive release of superoxide anion has been shown to contribute to postreperfusion oxidative damage in several ischemic organs [11, 12]. Increased thiobarbituric acid reactive substances (TBARSs), which is a marker of lipoperoxidation, has been observed in critically ill patients in association with multiorgan failure (MOF) development [7, 13].

Most studies about oxidative stress in sepsis have looked at a single time-point, usually at admission. In addition, very few of these studies have looked specifically at septic shock patients [14, 15]. But septic shock has distinctive features which may differ substantially from less severe forms of sepsis. It has a very dynamic course with several changes taking place simultaneously in just a few hours. Therefore, more comprehensive studies looking at the temporal evolution of oxidative stress in septic shock patients are required. This is a critical step to define windows of opportunity for interventions aimed at modulating this response.

Our main objective was to evaluate broadly the temporal profile of antioxidants and oxidative damage during septic shock evolution. Secondary objectives were to determine if oxidative damage markers and antioxidant levels are related to septic shock severity and to evaluate methionine sulfoxide, a novel marker of protein oxidative damage.

## 2. MATERIAL AND METHODS

This was a prospective observational study that included patients with diagnosis of septic shock admitted to a medical-surgical ICU of a university hospital from May 2004 to August 2005. The study was approved by the Ethical Committee of the Facultad de Medicina of the Pontificia Universidad Católica de Chile, Santiago, Chile, and an informed consent was obtained from all patients or their relatives.

### 2.1. Patients and management

Patients were enrolled if they fulfilled the following criteria: (1) diagnosis of septic shock according to the consensus conference [16], (2) less than 24 hours elapsed since admission to the emergency department, and (3) age older than 18 years. Exclusion criteria were (1) patients extremely ill, in whom survival interval was expected to be less than 48 hours, (2) use of any external vitamin supplement within one week before enrollment, (3) onco-hematological, and (4) chronic renal failure patients. All patients were managed according to a standard hemodynamic and respiratory algorithm [17].

### 2.2. Data collection

Demographic data, diagnosis, the acute physiology and chronic health evaluation (APACHE) II score, sepsis-related organ failure assessment (SOFA) score, hemodynamic and respiratory parameters, maximal vasoactive drug dose, general biochemistry (renal, hematological, and hepatic function), and C reactive protein (CPR) levels were registered at admission, 24 and 72 hours, as well as the seventh day of evolution.

### 2.3. Oxidative stress evaluation

Oxidative stress markers employed were thiobarbituric acid reactive substances (TBARSs), an index of lipid peroxidation, plus carbonyls, and methionine sulfoxide in plasma proteins as markers of protein oxidative damage. Antioxidant activity was evaluated by measuring: (a) total antioxidant capacity (TAC) determined with two methods: (1) total radical- trapping antioxidant potential (TRAP) and (2) ferric/reducing antioxidant power (FRAP); (b) nonenzymatic antioxidants: vitamins C and E, beta carotene, and lycopene; (c) enzymatic antioxidant cofactors: reduced and total red blood cell glutathione; and (d) nonspecific antioxidants: uric acid, bilirubin, and albumin. Blood samples were obtained at diagnosis (T0), 24 hours (T1), 72 hours (T3), and at the seventh day of evolution (T7). Delayed measurements were made in all survivors 3 months later. Normal values for each parameter were obtained from a group of 17 healthy volunteers matched for age and sex with septic shock patients.

### 2.4. Laboratory technique

All samples were stored at −20°C and were analyzed within 10 days, but for glutathione analysis, samples were stored with ACD at 4°C and were also analyzed within 10 days.

#### 2.4.1. Total antioxidant capacity (TAC)

**TRAP (total-trapping radical [Peroxyl] antioxidant potential)**

One mL of a mixture of Luminol (60 *μ*M)-AAPH (10 mM), in glycine buffer 50 mM pH: 9.40, kept in ice and darkness, was preincubated at 37°C. This mixture was poured into a cuvette of polystyrene (Clinicon) and placed in a luminometer (BioOrbit 1250, Finland) kept at 37°C. When the light emission reached a steady-state condition, 10 *μ*L of plasma or standard (Trolox) were added. The luminescence was plotted against the elapsed time. The induction time (tsample) was equated to the time required for the chemiluminescence intensity to reach a level equal to 40% of the intensity measured prior to the sample addition [18]. The quenching of the light emission was recorded in a Kipp & Zonen BD 111 recorder. The coefficient of variation for this measurement is 4, 04%.

**FRAP (ferric/reducing antioxidant power)**

Reduction at low pH of a ferric tripyridyltriazine (FeIII-TPTZ) complex to the ferrous form, by electron-donating antioxidants [19]. Samples were incubated in mixture of TPTZ, FeCl_3_, and acetate buffer (1 : 1 : 10) for 5 minutes at 37°C and quantified at 593 nm. The coefficient of variation for this measurement is 2,5%.

#### 2.4.2. Vitamin E (*α*-tocopherol), *β*-carotene, and lycopene

Lipid soluble antioxidant concentrations were determined by HPLC with electrochemical detection [20]. Plasma samples were precipitated with methanol and extracted with hexane and centrifuged. The organic phase was dried, and the residue was redissolved in methanol: ethanol. An isocratic phase reverse chromatography was performed using a Supelcosil C-8 column, and 20 mM LiClO_4_ in methanol: H_2_O (97.5 : 2.5) as mobile phase. For electrochemical detection, an amperometric BAS LC4C detector (Bioanalytical Systems Inc., West Lafayette, Ind, USA) was used with an applied potential of +0.6 V. The coefficient of variation for vitamin E is 1, 8%, for *β*-carotene is 9,3%, and for lycopene is 8,2%.

#### 2.4.3. Ascorbic acid

Determinations were carried out by a method based on the reduction of ferric chloride by ascorbic acid, with the resulting ferrous ion quantified by the addition of 2-,4-,6-tripyridyl-s-triazine [21]. Heparinized plasma samples were precipitated with trichloroacetic acid. The tubes were centrifuged at 20,800 g for 30 seconds. An aliquot from the supernatant was incubated with TPTZ and FeCl_3_ in buffer acetate for 5 minutes at 25°C, and quantified at 595 nm. The coefficient of variation for this measurement is 3,4%.

#### 2.4.4. Glutathione

**Total red blood cell glutathione**

Red blood cells were lysed and precipitated by adding perchloric acid 0.28 M to fresh blood anticoagulated with ACD. An aliquot from the supernatant, neutralized with K3PO4 1.75 M, was incubated with phosphate buffer 0.1 mM (EDTA 1 mM) and 5.5′-dithiobis (2-nitrobenzoic acid) 0.5 mM (sodium Citrate 1% p/v) for 7 minutes at 25°C and quantified at 412 nm [22].

**Reduced red blood cell gluthatione**

The cells were rinsed four times with 3 mL of ice cold PBS. Collected cells were diluted in phosphate buffer containing diethylenetriaminepentaacetic acid 1.34 mM pH 7.8. An aliquot was derivatized by the addition of N-(1-pyrenyl) maleimide 1.0 mM in acetonitrile. Solutions were incubated for 5 minutes at room temperature and then acidified with HCl to pH 2.5. An isocratic phase reverse chromatography was performed using a Supelcosil C-18 column and 65% acetonitrile: 35% H_2_O, 1 mL/liter acetic acid, and 1 mL/liter O-phosphoric acid as mobile phase. A fluorescence spectrophotometer detector (Merck-Hitachi F-1000, Darznstadt, Germany) was used for detection *λ*_ex_ 330 nm and *λ*_em_ 375 nm [23].

#### 2.4.5. TBARS

The method is based on the formation of an adduct TBA-MDA (2 : 1). Plasma samples, TBA solution (0.67%/NaOH 0.05N), and TCA 50% solution were placed in this order into a screw-cap test tube and incubated at 90°C for 45 minutes. The aqueous phase was quantified at 532 nm. TBARS plasmatic levels are expressed in micromolar equivalent MDA [24].

#### 2.4.6. Carbonyls

Plasma protein samples were reacted with dinitrophenylhydrazine and then adsorbed to wells of an ELISA plate, overnight at 4°C, before probing with a commercial antibody raised against protein-conjugated DNP. The biotin-conjugated primary antibody (anti-DNP Polyclonal IgG) was then reacted with streptavidin-conjugated horseradish peroxidase for quantification with TMB. Acidic stop solution was added, and absorbance measured at 450 nm. The method was calibrated using oxidized and reduced albumin [25]. The coefficient of variation for this measurement is 16, 2%.

#### 2.4.7. Methionine sulfoxide

Protein methionine sulfoxide content was measured by HPLC-fluorometric detection, using a modification of the method of Morgan et al. [26] and was expressed as the fraction of total methionine oxidized to methionine sulfoxide: proteins from 10 *μ*L plasma aliquots were precipitated with 60 *μ*L of ice-cold acetonitrile and centrifuged at 10000 g for 20 minutes; the protein pellets were hydrolyzed using methanesulfonic acid at 150°C for 90 minutes and were derivatized with o-phthaldialdehyde/*β*-mercaptoethanol solution. Derivatized amino acids were detected by an HPLC-fluorescence spectrophotometer detector (Merck-Hitachi F-1000), *λ*_ex_ 340 nm, and *λ*_em_ 455 nm, and quantified using standards containing methionine, methionine sulfoxide, and methionine sulfone. Analysis was performed by reverse-phase HPLC using a Synergy 4 *μ*M fusion column with a continuous gradient of mobile phase 75 mM Na acetate buffer pH 4.5: methanol: tetrahydrofuran (80 : 19 : 1) initial to (20 : 80 : 0) final, in 40 minutes. The coefficient of variation for this measurement is 9.2%.

### 2.5. Statistical analysis

Changes along time for the different oxidative stress markers were analyzed with linear mixed effects models. In addition, measurements in septic shock patients at each time point were compared with normal values from healthy matched subjects by a *t*-test for independent samples (samples exhibited a normal distribution). Correlations between severity parameters and oxidative stress markers were made with Pearson. Linear mixed effects models, adjusted by sex and age, were also used to confirm the association between severity parameters and oxidative stress. Results are expressed as mean ± SD, and a *P* < .05 was considered significant. The proc MIXED of the SAS statistical program was used for analysis.

## 3. RESULTS

A total of 21 patients fulfilled inclusion/exclusion criteria and were studied. Average age for all septic shock patients was 60 ± 20 years, APACHE II and SOFA scores at admission were 22.5 ± 6.6 and 11 ± 4.2, respectively. The main causes of septic shock were respiratory and abdominal. Only 4 patients died (19%). Demographic characteristics of the patients are summarized in Table 1.

### 3.1. Oxidative damage

**Figure 1:**
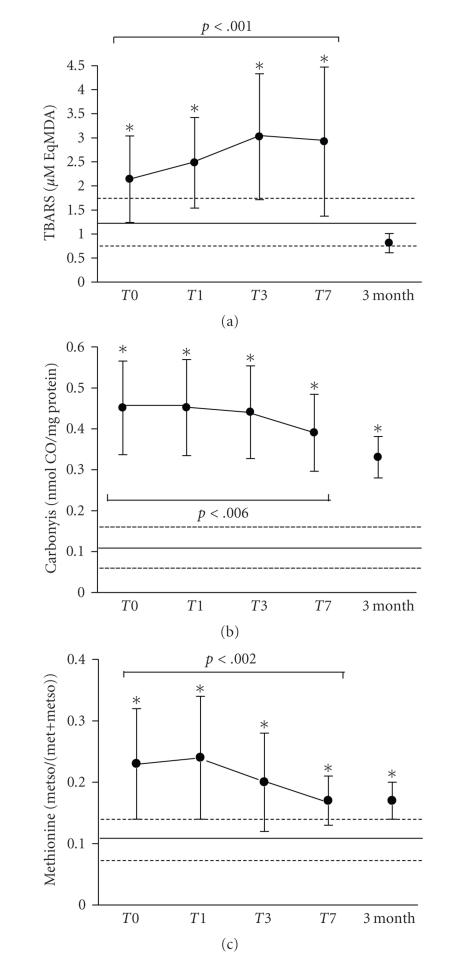
Temporal evolution of oxidative damage of all septic shock patients in (a) lipoperoxidation (TBARS), (b) carbonyls, and (c) Methionine sulfoxide. Normal values (mean ± SD) obtained from matched healthy subjects are shown as continuous and dotted lines, respectively. *p*: reflects variation in time calculated by linear mixed effects model.*Indicates significant differences between normal values and patients at different time points (*t*-test for independent samples).

At admission, all septic shock patients presented evidence of oxidative damage on lipids and proteins, measured either by carbonyls or methionine sulfoxide (Figure 1). TBARS levels were increased twofold at admission compared to normal values and increased further during the first week of evolution (*P* < .001) (Figure 1(a)). At 3 months, TBARS levels had normalized in septic shock survivors. Oxidative damage on proteins was also present at admission. Carbonyls were increased 4.5-fold and methionine sulfoxide 2.1-fold compared to normal values (Figures 1(b) and 1(c)). Thereafter, both markers tended to decrease during the first week of evolution (*P* < .01 for both), however they did not reach normal levels, even after three months.

### 3.2. Antioxidant activity

#### 3.2.1. Total antioxidant capacity (TAC) 

TAC measured by TRAP was normal at admission and day 1 (354 ± 123 versus 361 ± 50 *μ*M EqTlx for normal values), but decreased after day 3 (246 ± 110 *μ*M EqTlx, *P* < .001). At three months, TRAP levels remained decreased (279 ± 48 *μ*M EqTlx, *p* < 0.01). TAC levels measured by FRAP were elevated at admission (1656 ± 498 versus 1120 ± 47 *μ*M EqFe^2+^ for normal values, *P* < .001) and remained increased throughout the study period.

#### 3.2.2. Individual antioxidants

**Figure 2:**
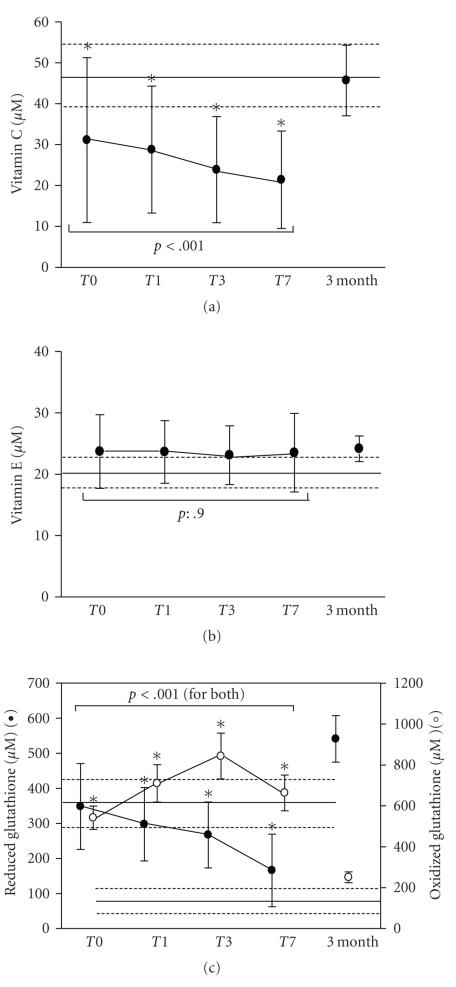
Evolution of antioxidant levels in all septic shock patients of (a) Vitamin C, (b) Vitamin E (alpha-Tocopherol), and (c) reduced and oxidized glutathione. Normal values (mean ± SD) obtained from matched healthy subjects are shown as continuous and dotted lines, respectively. *p*: reflects variation in time calculated by linear mixed effects model.*Indicates significant differences between normal values and patients at different time points (*t*-test for independent samples).

Vitamin C, beta carotene, and lycopene levels were significantly decreased at admission and continued to decrease progressively throughout the first week of evolution (*P* < .001) (Figure 2(a) for vitamin C). In contrast, vitamin E levels remained normal at all time points (Figure 2(b)). At 3 months, vitamin C and lycopene normal levels were recovered, but not yet beta carotene.

Reduced glutathione levels were normal at admission, but decreased after 24 hours and during the first week of evolution (*P* < .001). In contrast, total red blood cell glutathione levels were increased three-fold at admission and significantly increased during the first week of evolution (*P* < .001). At 3 months, both parameters had returned to normal values (Figure 2(c)).

**Figure 3:**
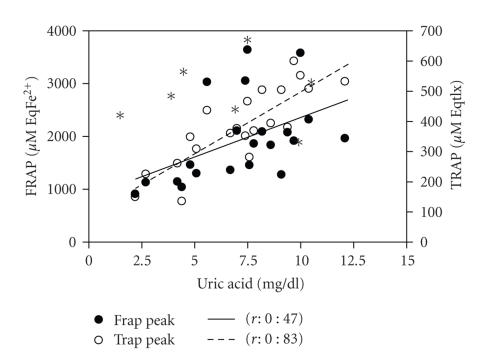
Correlations of peak FRAP and TRAP levels with peak uric acid levels. *P* < .05 for both correlations exposed.

Uric acid levels were increased at admission (6.7 ± 2.3 versus 5 ± 0.5 mg/dL for normal values, *P* < .01), but returned progressively to normal values within the first week. Bilirubin levels were also increased at admission (2.3 ± 2.1 versus 0.8 ± 0.5 mg/dL for normal values, *P* < .001) and did not change during evolution. A positive correlation between uric acid levels and TRAP and FRAP levels was observed (*r*: 0.83 and 0.47, resp., *P* < .03 for both) (Figure 3) and also between bilirrubin levels and FRAP levels (*r*: 0.75, *P* < .4 with FRAP, *P* < .001).

### 3.3. Oxidative stress and severity scores

SOFA scores decreased significantly along time in survivors, mainly due to a rapid decrease in cardiovascular and respiratory components of SOFA scores. Patients who died had higher levels of APACHE II and SOFA scores at admission and peak and also had higher lactate levels and peak doses of vasoactive drugs (*P* < .05 for all). C-reactive protein (CRP) levels were elevated at admission (23.5 ± 10.4 mg/dL versus 0.6 ± 0.3 for normal values, *P* < .001) and decreased during septic shock evolution, but persisted still elevated at day 7. Their levels were normal at 3 months.

### 3.4. At admission

**Figure 4:**
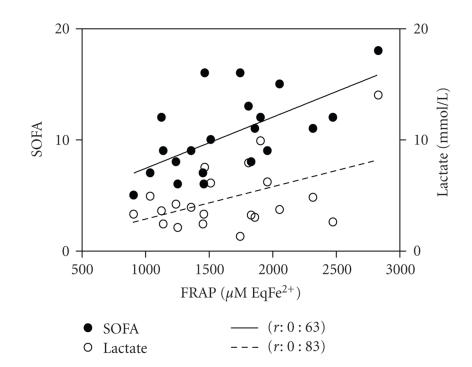
Admission correlations of peak SOFA score and peak lactate levels with peak FRAP levels. *P* < .05 for the correlation exposed.

A positive correlation of admission SOFA score with FRAP (Figure 4), vitamin C, and uric acid admission levels was observed (*r*: 0.63, *r*: 0.48, and *r*: 0.48, resp., *P* < .05 for all). APACHE II score was correlated with FRAP, TRAP, B-carotene, and uric acid admission levels (*r*: 0.5, *r*: 0.6, *r*: 0.5, and *r*: 0.56, resp., *P* < .05 for all), whereas lactate admission levels were correlated with FRAP (Figure 4) and bilirubin admission levels (*r*: 0.48 and 0.64, *P* < .05 for both). A negative correlation was observed between oxygenation (PaO_2_/FiO_2_ ratio) and FRAP, TRAP, vitamin C, and uric acid levels at admission (*r*: −0.44, *r*: −0.68, *r*: −0.58, and *r*: −0.48, resp., *P* < .05 for all). No significant correlations were observed between lipid or protein oxidative damage and severity of illness at admission.

### 3.5. During the evolution of shock

**Figure 5:**
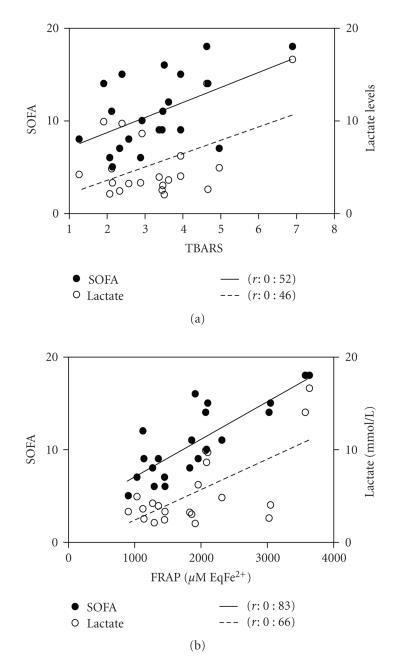
Evolution correlations of peak SOFA score and peak lactate levels with (a) peak TBARS levels and (b) peak FRAP levels. *P* < .05 for all correlations exposed.

TBARS peak levels exhibited a positive correlation with peak SOFA score and peak lactate levels (*r*: 0.52 and *r*: 0.46, *P* < .05 for both) (Figure 5(a)). TBARS peak levels were also positively correlated with peak FRAP (*r*: 0.57, *P* < .05). No correlations or associations were observed between protein oxidative damage and antioxidant consumption or severity of disease at admission or during septic shock evolution.

Peak SOFA score was also correlated with peak levels of FRAP (Figure 5(b)), TRAP, bilirubin, and vitamin C levels (*r*: 0.82, *r*: 0.82, *r*: 0.6, and *r*: 0.56, resp., *P* < .05 for all). APACHE II score was correlated with peak levels of FRAP, TRAP, uric acid, and vitamin C levels (*r*: 0.67, *r*: 0.66, *r*: 0.45, and *r*: 0.6, resp., *P* < .05 for all). Peak lactate levels were correlated with FRAP (Figure 5(b)) and bilirubin peak levels (*r*: 0.66 and *r*: 0.65, *P* < .05 for both). All correlations presented above were confirmed as positive associations by linear mixed effects model in time.

## 4. DISCUSSION

Our study prospectively evaluated the temporal profile of antioxidants and oxidative damage markers during the first week of evolution of septic shock and their correlation with severity. We found evidence of early oxidative damage on proteins and lipids associated with severe vitamin C, *β*-carotene, lycopene, and reduced glutathione depletion, but not associated with reduced TAC or vitamin E levels. Early production of reactive oxygen species has been documented in endotoxin models [14], and clinical studies also have found that critical care patients already present evidence of oxidative stress at admission [2, 7–9, 27, 28]. Therefore, oxidative damage is a rather early phenomenon of the systemic inflammatory response, including septic shock, taking place even before septic patients arrive to critical care units. This notion may in part explain why interventions which have been shown to be effective to prevent oxidation [29] may not be effective to revert an already established oxidative damage in the ICU setting.

We also found that lipoperoxidation increases along time. This may be the result of an initial insult, most probably ischemic, followed by an independent perpetuating process, which could be explained by the autocatalytic nature of the lipoperoxidation cascade, by persistent immune activation, by continuous episodes of reperfusion, or by insufficient or ineffective antioxidation. Protein oxidative damage, in contrast, significantly decreases along time in parallel with the decrease of SOFA score. Decreasing levels of protein oxidative damage have been described before [30] and since we expressed protein oxidative markers as a proportion of total plasma proteins, changes in carbonyls and methionine sulfoxide levels should not be influenced by changes in total body proteins in our study and may truly reflect that protein oxidative damage is maximal early in the septic process.

Lipoperoxidation was positively correlated to septic shock severity and to organ dysfunction estimated by SOFA, suggesting that oxidative damage may have a role in the development of multiorgan dysfunction. Recently, Motoyama et al. [8] in SIRS patients also found that higher levels of TBARS at admission and in time were associated with the development of multiorgan failure (MOF). In contrast, we did not find any significant correlation between protein oxidative damage and septic shock severity at any time point. Since several critical proteins have been described to be partially or completely inactivated by the action of reactive oxygen species during sepsis, suggesting that oxidative damage over proteins could have a role in sepsis [31, 32], it is possible that carbonyls and methionine sulfoxide, although sensitive markers of protein damage [33, 34], do not reflect the real impact of oxidative stress over critical proteins and enzymes. 

TAC levels exhibited different kinetics along time according to the method used to estimate them. These differences could be explained looking at the individual contributors to serum TAC levels, which are represented in different proportions according to the method used to estimate TAC. Particularly in septic shock, TRAP levels are strongly influenced by uric acid levels (*r*: 0.83), explaining the parallel decrease of both markers along time. In contrast, FRAP levels are less dependent on uric acid, but may be also influenced by bilirubin and other nonidentified compounds, which may explain the increase in FRAP levels at admission and during the study period [35]. Both parameters, TRAP and FRAP and their major contributors, uric acid and bilirubin, were positively correlated with septic shock severity. Previous studies have already suggested that TAC levels may be higher in more severe forms of sepsis [15, 35, 36]. In particular, elevations of uric acid during sepsis can be explained by an increase in uric acid production associated with activation of Xantine oxidase (XO) in ischemic territories during sepsis and septic shock [2].

Vitamin C levels were below normal values during the study period, probably explained by ongoing consumption. Although we expected to observe larger consumption in the most severe patients, we found the opposite; more severe septic patients had higher vitamin C levels. We speculate that lower consumption of vitamin C observed in most severe patients might be explained by larger availability of other nonenzymatic antioxidants in these patients, namely, uric acid and bilirubin, both of which may act as effective antioxidants in plasma [37, 38]. Interestingly, even though most severe patients exhibited higher TAC and vitamin C levels, these were also positively correlated to peak TBARS indicating that lipoperoxidation may occur despite normal or even increased antioxidant capacity.

At 3 months, lipoperoxidation levels normalized in septic shock survivors, whereas protein oxidative markers still remained elevated. Persistent elevation of carbonyls and methionine sulfoxide is difficult to explain. Ongoing oxidative stress is unlikely since CRP levels were normal at three months, and most patients were already doing well at home. A slow protein turnover would explain that 3 months were insufficient to reach normal levels of these markers [34]. This finding suggests that these indexes could be used as markers of a previous oxidative stress situation.

A limitation of our study was the exclusion of some patients that were extremely ill and were expected to die before 48 hours, patients admitted from the ward and chronic renal failure patients. These patients may present a different antioxidant—oxidative scenario. However, during the study period, from all consecutive septic shock patients admitted to our unit only 5 were excluded. These exclusion criteria may partially explain the 19% mortality, which is lower than predicted from SOFA and APACHE II scores. Our reported mortality for septic shock is 33% [17].

## 5. CONCLUSIONS

Our study shows early and persistent oxidative stress during septic shock, reflected on lipid and protein damage and on nonenzymatic antioxidant apparent consumption. Protein oxidation reaches its peak early at admission, but lipoperoxidation continues to increase during the first days of evolution. More severe patients exhibit higher levels of lipoperoxidation but also higher levels of total antioxidant capacity, uric acid, bilirubin, and vitamin C. We showed for the first time that methionine sulfoxide levels are increased in septic shock patients and, therefore, may be used as a sensitive marker of protein oxidation in sepsis.

## Figures and Tables

**Table 1:** Characteristics of the patients.

Age (years)	60.2 ± 20.7
Gender (female/male)	10/11
APACHE II score	22.5 ± 6.6
SOFA score at admission (T0)	10.5 ± 3.6
24 hours (T1)	9.7 ± 4
72 hours (T3)	7.5 ± 4.7
7th day (T7)	5.5 ± 6.8
Septic shock etiology (*n* (%))	
Pneumonia	6 (35.3)
Abdominal	7 (29.4)
Urological	4 (11.8)
Others	4 (23.5)
C-Reactive protein levels at admission	23.5 ± 10.4
Lactate levels at admission (mmol/l)	4.8 ± 3
Noradrenaline (Max. dose (ug/kg/min))	0.31 ± 0.2
ALI/ARDS (*n* (%))	19 (88)
Mechanical ventilation (*n* (%))	16 (70)

ALI/ARDS, Acute lung injury/Acute respiratory distress syndrome; APACHE II score, Acute physiology and chronic health evaluation score II on ICU admission; SOFA score, Sequential organ failure assessment. Values reported are mean ± SD or *n* (%).
